# Central Adrenal Insufficiency Is Rare in Adults With Prader–Willi Syndrome

**DOI:** 10.1210/clinem/dgaa168

**Published:** 2020-03-31

**Authors:** Anna G W Rosenberg, Karlijn Pellikaan, Christine Poitou, Anthony P Goldstone, Charlotte Høybye, Tania Markovic, Graziano Grugni, Antonino Crinò, Assumpta Caixàs, Muriel Coupaye, Sjoerd A A Van Den Berg, Aart Jan Van Der Lely, Laura C G De Graaff

**Affiliations:** 1 Internal Medicine, Division of Endocrinology, Erasmus MC, University Medical Center Rotterdam, Rotterdam, The Netherlands; 2 Assistance Publique-Hopitaux de Paris, Nutrition Department, Institute of Cardiometabolism and Nutrition, Pitie-Salpetriere Hospital, Sorbonne Universite, Paris, France; 3 International Network for Research, Management & Education on Adults with PWS; 4 European Reference Network on Rare Endocrine Conditions; 5 PsychoNeuroEndocrinology Research Group, Neuropsychopharmacology Unit, Division of Psychiatry, Computational, Cognitive and Clinical Neuroimaging Laboratory, Department of Brain Sciences, Faculty of Medicine, Imperial College London, Hammersmith Hospital, London, UK; 6 Department of Molecular Medicine and Surgery, Patient Area Endocrinology and Nephrology, Inflammation and Infection Theme, Karolinska Institute and Karolinska University Hospital, Stockholm, Sweden; 7 Metabolism & Obesity Services, Royal Prince Alfred Hospital, Camperdown, Australia; 8 Boden Collaboration, University of Sydney, Sydney, Australia; 9 Divison of Auxology, Italian Auxological Institute, IRCCS, Piancavallo, Italy; 10 Reference Center for Prader–Willi Syndrome, Bambino Gesu Hospital, Research Institute, Palidoro (Rome), Italy; 11 Department of Endocrinology and Nutrition, Hospital Universitari Parc Taulí (UAB), Institut d’Investigacio i Innovacio Parc Taulí (I3PT), Sabadell, Spain; 12 Department of Clinical Chemistry, Erasmus MC, University Medical Center Rotterdam, Rotterdam, The Netherlands; 13 Academic Center for Growth, Erasmus MC, University Medical Center Rotterdam, Rotterdam, The Netherlands

**Keywords:** Prader–Willi syndrome, central adrenal insufficiency, hypocortisolism, insulin tolerance test, metyrapone test

## Abstract

**Context:**

Prader–Willi syndrome (PWS) is associated with several hypothalamic-pituitary hormone deficiencies. There is no agreement on the prevalence of central adrenal insufficiency (CAI) in adults with PWS. In some countries, it is general practice to prescribe stress-dose hydrocortisone during physical or psychological stress in patients with PWS. Side effects of frequent hydrocortisone use are weight gain, osteoporosis, diabetes mellitus, and hypertension—already major problems in adults with PWS. However, undertreatment of CAI can cause significant morbidity—or even mortality.

**Objective:**

To prevent both over- and undertreatment with hydrocortisone, we assessed the prevalence of CAI in a large international cohort of adults with PWS. As the synacthen test shows variable results in PWS, we only use the metyrapone test (MTP) and insulin tolerance test (ITT).

**Design:**

Metyrapone test or ITT in adults with PWS (N = 82) and review of medical files for symptoms of hypocortisolism related to surgery (N = 645).

**Setting:**

Outpatient clinic.

**Patients or Other Participants:**

Eighty-two adults with genetically confirmed PWS.

**Main Outcome Measure:**

For MTP, 11-deoxycortisol > 230 nmol/L was considered sufficient. For ITT, cortisol > 500 nmol/L (Dutch, French, and Swedish patients) or > 450 nmol/L (British patients) was considered sufficient.

**Results:**

Central adrenal insufficiency was excluded in 81 of 82 patients. Among the 645 patients whose medical files were reviewed, 200 had undergone surgery without perioperative hydrocortisone treatment. None of them had displayed any features of hypocortisolism.

**Conclusions:**

Central adrenal insufficiency is rare (1.2%) in adults with PWS. Based on these results, we recommend against routinely prescribing hydrocortisone stress-doses in adults with PWS.

Prader–Willi Syndrome (PWS) is a rare and complex genetic disorder caused by the lack of expression of paternally inherited genes in the PWS region on chromosome 15q11-q13 (1). Apart from intellectual disability, sleep-related disorders and hypotonia, PWS is associated with hypothalamic dysfunction (1, 2), resulting in an insatiable appetite, disturbed thermoregulation, abnormal pain perception, and pituitary hormone deficiencies (2–4). In adults with PWS, growth hormone (GH) deficiency is reported in 0–38% (5, 6) and hypothyroidism in 13.6% (7) of patients. Hypogonadism is present in the majority of patients with PWS and can be either primary or central (8). There is no agreement on the prevalence of deficiencies in other pituitary hormones.

Mortality is high among patients with PWS (3% annual death rate across all ages) and death is often unexpected (9). It has been suggested that sudden death in patients with PWS might partly be explained by central adrenal insufficiency (CAI): an inadequate (increase in) cortisol production by the adrenal glands due to the insufficient secretion of adrenocorticotropic hormone (ACTH) or corticotropin-releasing hormone (CRH) by the pituitary gland or hypothalamus, respectively. If left untreated, CAI can result in an adrenal crisis, which is life-threatening. During crisis, a drop in blood pressure, organ failure, and/or mental alteration can lead to hospitalization or even treatment in the Intensive Care Unit.

Replacement with synthetic cortisol (hydrocortisone) is therefore advocated if patients have symptoms of CAI (10), which include muscle weakness, fatigue, and weight loss. However, these symptoms are unreliable in PWS. Muscle weakness and fatigue are common in PWS (11) and weight loss is not unusual, as most individuals with PWS are on a diet. In some countries, it is general practice to administer hydrocortisone during stressful situations, such as surgery, illness, or intense psychological stress (10, 12). However, stress and illness are often hard to define in individuals with PWS, as hypothalamic dysfunction reduces pain perception and the ability to mount a fever (11). Furthermore, the behavioral phenotype of PWS is characterized by frequent temper outbursts, causing psychological stress.

These uncertainties lead to frequent administration of hydrocortisone in people with PWS. Side effects of frequent overuse of hydrocortisone are weight gain, osteoporosis, diabetes mellitus, and hypertension (13), already major problems in adults with PWS (14). Ideally, hydrocortisone should only be prescribed when it is absolutely necessary.

There is no agreement on the prevalence of CAI and on the need for hydrocortisone use in adults with PWS, due to the use of different test methods and the fact that most studies involved children, not adults (10, 12, 15–21). In a previous Dutch study among 25 children with PWS, 15 (60%) were diagnosed with CAI based on ACTH levels during single-dose metyrapone tests (sMTP) using an ACTH cutoff < 33 pmol/L (12). However, the use of ACTH levels in the evaluation of the hypothalamic-pituitary-adrenal (HPA) axis has been debated, as it can lead to false-positive results (22). Studies using other test methods to diagnose CAI found much lower prevalences or even total absence of CAI in children (10, 16–21) and adults (15, 18). However, most studies used the synacthen test, which is adequate for diagnosing primary adrenal insufficiency (PAI) but less reliable for diagnosing CAI (23).

As both untreated CAI and overtreatment with hydrocortisone can have severe adverse consequences for the patient, it is important to know the true prevalence of CAI in adults with PWS. National PWS experts from 7 countries have collaborated to define the prevalence of CAI in 82 adults with PWS, which is a large group for such a rare disorder. As the use of less sensitive diagnostic tests causes uncertainty, we only report the results from the 2 most robust tests for diagnosis of CAI: the insulin tolerance test (ITT) and multiple-dose metyrapone test (MTP). Apart from collecting MTP and ITT data, we reviewed the medical records of 645 adults with PWS to define the true prevalence of CAI in adults with PWS.

## Methods

All participating centers obtained approval from ethics committees and/or individual patients to retrospectively collect data on the ITT and MTP performed in adults with PWS.

### Part A: diagnosis of CAI

The HPA axis was tested in 56 Dutch, 10 French, 10 British, and 6 Swedish adults with PWS as part of regular patient care. Part of the data on the 6 Swedish patients has been published previously (18). Eight adults with PWS did not undergo ITT/MTP, because they used daily hydrocortisone based on synacthen test failure or extremely low baseline cortisol. Although diagnosis of CAI was not based on MTP or ITT, the patients were not retested by MTP or ITT due to behavioral issues or for other patient-related reasons.

#### Metyrapone test procedure.

Patients were hospitalized for 2 consecutive days. On day one, after a 10- to 12-hour overnight fast, blood samples for ACTH and cortisol were taken at 7:45 am, and metyrapone (750 mg, Laboratoire HRA Pharma, Paris, France) was administered orally at 8:00 am, 12:00 pm, 4:00 pm, 8:00 pm, 12:00 am, and 4:00 am. Patients were fed at 6:00 pm. On day 2, blood samples for cortisol and 11-deoxycortisol were taken at 7:45 am after a 10- to 12-hour overnight fast. Blood samples were taken through a peripheral intravenous catheter. Patients were recumbent from 7:00 am until the blood collection was completed. To ensure appropriate cortisol suppression, we used a day 2 morning cortisol cutoff of 200 nmol/L.

To assess the clinical value of ACTH during MTP in the diagnosis of CAI, ACTH was also measured. Delta ACTH was defined as the difference between ACTH at the start of the test (baseline ACTH) and the peak ACTH level.

#### Insulin tolerance test procedure.

Patients were hospitalized for 1 day. After a 10- to 12-hour overnight fast, short-acting insulin (Insuman Rapid®, Actrapid®, 0.15U/kg) was administered at t = 0 in order to achieve hypoglycaemia (blood glucose ≤ 2.2 mmol/L). Blood samples for cortisol and glucose were taken at t = 0, 30, 60, and 90 minutes through a peripheral intravenous catheter. In Dutch and British patients, ACTH was also measured. Additional insulin (dose based on the actual glucose level and weight of the patient) was administered if the glucose level was ≥ 2.2 mmol/L at t = 60, unless patients showed severe clinical signs of hypoglycaemia, and blood samples were taken at t = 80, 90, 100, 120, and 150 minutes. The patients were recumbent from the start of the study until the final blood sample was collected. If women were taking oral estrogens, these were stopped at least 6 weeks before the ITT to avoid artefactual elevations of measured cortisol due to increased levels of cortisol-binding globulin. Corticosteroids were ceased at least 1 week before testing, both for MTP and ITT.

Assays. Adrenocorticotropic hormone and cortisol levels were measured with Siemens Immulite 2000XPi (Dutch patients; British patients for all ACTH measurements and for cortisol before August 2010), chemiluminescence immunoassay Abbott Architect i2000 (British patients for cortisol measurements after August 2010, to which all British cortisol results were aligned based on a field comparison study), immunochimiluminescence Roche Cobas (French patients), or electrochemiluminescence immunoassay Elecsys, Roche (Swedish patients). Blood glucose was measured with Roche Cobas C (Dutch, French, and Swedish patients) and Abbott Architect i2000 (British patients). 11-deoxycortisol was measured with UPLC-MSMS (Waters TQS, Etten-Leur, the Netherlands) in all patients. For MTP, 11-deoxycortisol > 200 nmol/L (>230 nmol/L or 7.9 µg/dL in the Dutch center due to harmonization) was considered sufficient (24). For Dutch, French, and Swedish patients who underwent ITT, cortisol > 500 nmol/L (18.1 µg/dL) was considered sufficient, whereas for British patients cortisol > 450 nmol/L (16.3 µg/dL) was considered sufficient (after alignment of the previous > 500 nmol/L [18.1 µg/dL] cutoff from Siemens Immulite 2000 assay to the Abbott Architect i2000 assay). The reference range for baseline cortisol was 200–700 nmol/L.

### Part B: patient file review

We reviewed the medical files of all 645 adult patients with PWS who visited the centers participating in the International Network for Research, Management & Education on Adults with PWS: Italy (240), France (110), the Netherlands (110), Australia (60), Spain (45), Sweden (38), and the UK (42). We collected clinical data to determine rates and means of diagnosis of CAI, the number of patients on continuous hydrocortisone treatment, and the number of patients that underwent surgery with and without stress doses of hydrocortisone.

### Part C: literature review

We performed a PubMed search and reviewed the medical literature for studies that have assessed adrenal function in > 1 patient by dynamic testing of the HPA axis. We used the following search strategy: “Prader–Willi Syndrome” [Mesh] AND “adrenal” [All Fields].

### Data analysis

Data were analyzed with R version 3.6.0. Continuous data are presented as median (range) and categorical data are presented as count. We calculated Spearman’s rho for the analysis of correlations. *P*-values of < 0.05 were considered significant.

### Role of the funding source

For this study, we received financial support from CZ fund. CZ fund had no role in the study design; in the collection, analysis, and interpretation of data; in writing the report; or in the decision to submit the paper for publication.

## Results

Eighty-two patients (46 males, 36 females) were tested for CAI. Forty-six patients underwent MTP and 36 patients underwent ITT. None of the patients underwent both tests. Patient characteristics are shown in Table 1.

**Table 1.** Characteristics of the study population

	ITT (n = 36)			MTP (n = 46)			Total (n = 82)		
Patients with PWS	Male	Female	All	Male	Female	All	Male	Female	All
N	19	17	36	27	19	46	46	36	82
Nationality									
British	5	5	10	0	0	0	5	5	10
Dutch	6	4	10	27	19	46	33	23	56
French	3	7	10	0	0	0	3	7	10
Swedish	5	1	6	0	0	0	5	1	6
Age (years)									
Median	25.0	24.0	24.9	28.0	22.5	25.3	25.9	23.5	25.1
Range	18.0–36.0	18.0–55.3	18.0–55.3	18.1–55.5	18.2–39.0	18.1–55.5	18.0–55.5	18.0–55.3	18.0–55.5
BMI (kg/m^2^)									
Median	28.3	32.0	30.3	27.4	31.5	28.4	28.2	31.7	29.1
Range	21.2–62.0	20.3–58.2	20.3–62.0	20.0–57.0	21.2–49.7	20.0–57.0	20.0–62.0	20.3–58.2	20.0–62.0
Genotype									
mUPD	2	5	7	10	8	18	12	13	25
DEL	9	9	18	16	10	26	25	19	44
ICD	1	0	1	0	0	0	1	0	1
mUPD or ICD	1	2	3	0	0	0	1	2	3
mDEL	1	0	1	0	0	0	1	0	1
Methylation-positive	5	1	6	1	1	2	6	2	8
GH ^i^ treatment during childhood	4	6	10	11	13	24	15	19	34
Current GH treatment	3	1	4	8	11	19	11	12	23

Abbreviations: BMI, body mass index; DEL, paternal deletion; GH, growth hormone, ICD, imprinting center defect; ITT, insulin tolerance test; mDEL, SNORD116 microdeletion; MTP, multiple-dose metyrapone test; mUPD, uniparental maternal disomy; PWS, Prader–Willi syndrome.

### Multiple-dose metyrapone test

**Figure 1.**
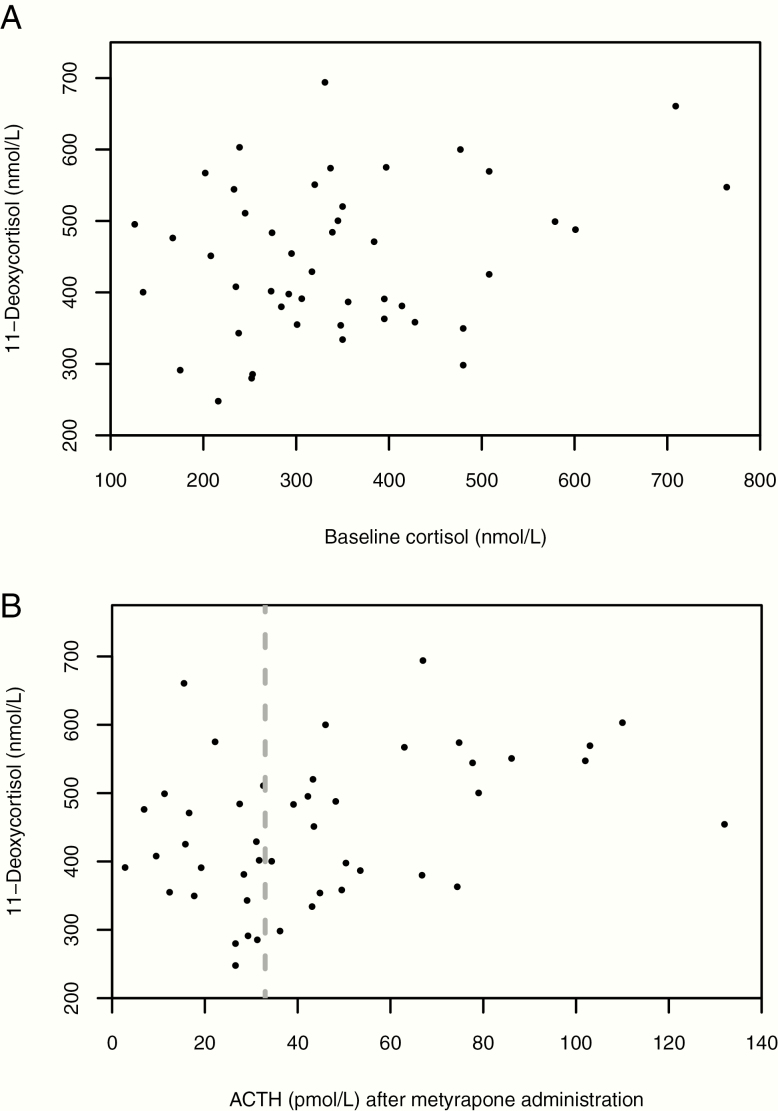
Results of the multiple-dose MTP in patients with Prader–Willi syndrome. N = 46. **A**: Relation between baseline cortisol (nmol/L) and 11-deoxycortisol (nmol/L). Spearman’s rho was 0.16 (*P* = 0.28). Even patients with low baseline cortisol had normal 11-deoxycortisol levels. **B**: Relation between ACTH (pmol/L) after metyrapone administration and 11-deoxycortisol (nmol/L). Spearman’s rho was 0.35 (*P* = 0.02). The dotted line represents the cutoff of 33 pmol/L used by the Dutch pediatric study (12), which would falsely classify 21 patients with sufficient increase in 11-deoxycortisol levels as “adrenal insufficient.”

The results of the MTP are shown in Table 2 and Fig. 1. All patients’ 11-deoxycortisol levels were above 230 nmol/L (median 440.1, range 247.8–694.0 nmol/L). In 2 patients, the day 2 morning cortisol was above the cutoff of 200 nmol/L, namely 213 and 211 nmol/L. Although this was suggestive of inadequate inhibition of 11-β hydroxylase, it still provoked an adequate increase of 11-deoxycortisol (298.2 and 425.3 nmol/L at day 2), confirming that the function of the HPA axis was normal.

**Table 2.** Results of the multiple-dose metyrapone test

	Before ^a^		After ^b^			Delta ^c^	CAI Cutoff ^d^
	ACTH (pmol/L)	Cortisol (nmol/L)	ACTH (pmol/L)	Cortisol (nmol/L)	11-deoxycortisol (nmol/L)	ACTH (pmol/L)	11-deoxycortisol (nmol/L)
Median	3.5	325.5	37.7	70.0	440.1	33.4	<230
Range	1.3–16.2	126.0–764.0	2.8–132.0	28.0–213.0 ^e^	247.8–694.0	-1.4–118.9	

Abbreviation: CAI, central adrenal insufficiency.

^a^ Before metyrapone administration. ^b^ After metyrapone administration. ^c^ Increase in ACTH after metyrapone administration. ^d^ 11-deoxycortisol cutoff for diagnosis of CAI. ^e^ In 2 patients, the day 2 morning cortisol was above the cutoff of 200 nmol/L, namely 213 and 211 nmol/L. Although this was suggestive of inadequate inhibition of 11-β hydroxylase, it still provoked an adequate increase of 11-deoxycortisol (298.2 nmol/L and 425.3 at day 2), showing function of the HPA axis was normal.

There was no significant relation between baseline cortisol and 11-deoxycortisol after metyrapone administration (*ρ* = 0.16; *P* = 0.28), as shown in Fig. 1A. All patients with a baseline cortisol below the lower reference limit of 200 nmol/L (lowest: 126.0 nmol/L) had a sufficient 11-deoxycortisol response.

The median (range) ACTH level after metyrapone administration was 37.7 (2.8–132.0) pmol/L. The ACTH level during MTP correlated poorly with 11-deoxycortisol level (*ρ = *0.35; *P* = 0.02; Fig. 1B), as did delta ACTH (*ρ* = 0.38; *P* = 0.01).

### Insulin tolerance test

The results of the ITT are shown in Table 3. Only 2 patients did not reach the target hypoglycaemia of ≤ 2.2 mmol/L as at near-target glucose levels (2.6 mmol/L in 1 patient and 2.4 mmol/L in the other); they already had clinical signs of severe hypoglycaemia (somnolence, reduced arousal, and increased perspiration) such that it was considered unethical to administer more insulin.

**Table 3.** Results of the insulin tolerance test

	Baseline Cortisol (nmol/L)	Peak Cortisol (nmol/L)	Glucose (mmol/L) ^a^	Baseline ACTH (pmol/L)	Peak ACTH (pmol/L)	Delta ACTH (pmol/L) ^b^	CAI Cutoff (nmol/L) ^c^
France (n = 10)							
Median	229.0	735.5	1.6	N/A	N/A	N/A	<500
Range	102.0–384.0	494.0–1021.0	0.6–2.2	N/A	N/A	N/A	
The Netherlands (n = 10)							
Median	233.0	702.0	1.9	3.3	61.2	57.1	<500
Range	119.0–502.0	530.0–883.0	1.4–2.4	1.1–6.2	23.8–93.5	21.2–90.5	
Sweden (n = 6)							
Median	185.5	722.5	1.7	N/A	N/A	N/A	<500
Range	175.0–265.0	502.0–822.0	1.2–2.6	N/A	N/A	N/A	
UK (n = 10)							
Median	172.5	522.5	1.5	3.7	N/A	N/A	<450
Range	93.0–545.0	455.0–971.0^d^	1.0–2.1	1.7–6.4	N/A	N/A	

Abbreviations: CAI, central adrenal insufficiency; N/A, not available.

^a^ Two patients had glucose levels of 2.4 mmol/L and 2.6 mmol/L, respectively. All other patients had glucose levels ≤ 2.2 mmol/L. ^b^ Increase in ACTH after insulin administration. ^c^ Peak cortisol cutoff for diagnosis of CAI. One French patient had peak cortisol < 500 nmol/L. d In the UK, the cutoff for CAI is 450 nmol/L (see also: methods).

During the ITT, 35 of 36 patients (including the 2 who did not reach hypoglycaemia of ≤ 2.2 mmol/L) had peak cortisol levels above the cutoff of 500 nmol/L. Only 1 French patient, who had no physical signs of CAI but was tested because of the transition from pediatric to adult care, had a suboptimal peak cortisol level of 494 nmol/L. He was prescribed hydrocortisone for use during physical stress. Since it was very difficult to obtain intravenous access in this patient, the ITT was not repeated.

The peak cortisol correlated poorly with peak ACTH (*ρ* = -0.04; *P* = 0.91) and delta ACTH during ITT (*ρ* = 0.05; *P* = 0.88).

### Reviewing medical files

We reviewed the medical files of 645 adult patients with PWS. Six French, 1 Australian, and 1 British patient used daily hydrocortisone based on a previous low morning cortisol, low-dose short synacthen test or high-dose short synacthen test. Two Dutch patients did allow to be retested, although they used daily hydrocortisone (1 based on a CRH test and the other after an event during surgery, which at that time was misinterpreted as an adrenal crisis). In these patients, hydrocortisone was successfully tapered and CAI was excluded by MTP.

As Dutch guidelines recommend the use of hydrocortisone during physical or psychological stress, even in patients without proven CAI, 30 of the 110 Dutch patients whose medical files were reviewed, received hydrocortisone during surgery (without performing an HPA function test first). Eighteen of these 30 subjects were subsequently formally tested for CAI (15 MTP; 3 ITT) and all of them were found to have sufficient HPA function, ie, no indication for perioperative hydrocortisone. Fifty-three Dutch patients had surgery without hydrocortisone, as they were operated before the guidelines were published. None of them had any complications during or after surgery. Twenty-six of these 53 subjects were subsequently tested for CAI (20 MTP; 6 ITT) and all were found to be sufficient. None of the 535 non-Dutch patients received hydrocortisone stress-dose during illness or surgery without undergoing an HPA function test (Table 4).

**Table 4.** Review of medical files of adult patients with Prader–Willi syndrome

Country	Patient Files Reviewed (N)	Surgery with HC (N)	Surgery without HC (N)	Adrenal Crisis During Surgery (N)
Italy	240	0	97	0
UK	42	0	13	0
Sweden	38	0	8	0
Spain	45	0	7	0
France	110	0	9	0
Australia	60	1 ^a^	13	0
The Netherlands	110	30 ^b^	53 ^c^	0
**Total**	645	31	200	0

Abbreviation: HC, hydrocortisone stress dose.

^a^ The patient had been using daily hydrocortisone after an insufficient low-dose synacthen test. ^b^ 2 patients had been using daily hydrocortisone but were later tested sufficient; 28 had been using hydrocortisone during operation (16 of them were later tested sufficient). ^c^ 26 patients later tested sufficient (20 multiple-dose metyrapone test, 6 insulin tolerance test).

In total, of the 645 patients whose files were reviewed, 200 underwent surgery without the administration of stress doses of hydrocortisone. None of them displayed any features of hypocortisolism or adrenal crisis.

Based on ITT and MTP, the prevalence of CAI in the 82 adults with PWS was 1.2%. Findings from our study and those from other groups (10, 12, 15–21) are detailed in Table 5.

**Table 5.** Summary of studies investigating the prevalence of central adrenal insufficiency in patients with Prader–Willi syndrome

Study	N	Median Age, Years (Range)	GH Treatment (%)	Testing Method	Prevalence (%)
Lind van Wijngaarden, et al (2008) (12)	25	9.7 (3.7–18.6)	100	sMTP	60
Connell, et al (2010) (17)	4	7.16 (0.43–16.27)	N/A	LDSST	4
	6			HDSST	
	15			ITT	
Nyunt, et al (2010) (16)	41	7.68 (±5.23) ^a^	46	LDSST	0
Farholt, et al (2011) (18)	58	22 (0.42–48.0)	62	HDSST	0
	8			ITT	0
Corrias, et al (2012) (10)	84	7.7 (±5.0) ^a^	63	LDSST	14.2
	9 ^b^			HDSST	4.8
Grugni, et al (2013) (15)	53	27.9 (18.0–45.2)	30	LDSST	15
	6 ^b^			HDSST	7.5
Beauloye, et al (2015) (21)	14	4.55 (0.8–14.7)	25	GT	5
	7 ^c^	5.6 (3.5–14.4)		ITT	
Obrynba, et al (2018) (19)	21 ^d^	13.9 (±10.9) ^a^	76	LDSST	29
				sMTP	0
Oto, et al (2018) (20)	36	2.0 (0.6–12.0)	0	ITT	0
This study (2019)	46	25.3 (18.1–55.5)	28	MTP	0
	36	24.9 (18.0–55.3)		ITT	2.8

Abbreviations: GT, glucose tolerance test; HDSST, high-dose synacthen test; ITT, insulin tolerance test; LDSST, low-dose synacthen test; MTP, multiple-dose metyrapone test; N/A, not available; sMTP, single-dose metyrapone test.

^a^ Age expressed as mean ± SD. ^b^ Number of subjects who failed the LDSST and underwent HDSST confirmation test. ^c^ 1 subject was tested by GT and ITT. ^d^ All subjects were tested by LDSST and sMTP.

## Discussion

We tested the HPA axis in 82 adult patients with PWS and conclude that CAI is very rare (1.2%) in adults with PWS. This low prevalence of CAI is in line with the majority of studies investigating CAI in people with PWS (10, 15–21) (Table 5) but is in sharp contradiction with the Dutch pediatric study by De Lind van Wijngaarden et al (12), who diagnosed CAI in 60% of Dutch children with PWS.

A likely explanation for the discrepancy between the Dutch pediatric study and the other studies investigating CAI in people with PWS is the difference in the type of provocative test used. The different types of provocative tests used for diagnosing CAI are described in the supplementary data (Table S2), which are located in a digital data repository (25).

In the Dutch pediatric study (12), the sMTP was used to assess the prevalence of CAI. Patients were considered as having CAI when postmetyrapone ACTH levels were < 33 pmol/L (150 pg/mL) (26). However, a Dutch reference range study (24) showed that ACTH levels during sMTP in healthy adult volunteers ranged from 9.2 to 211.0 pmol/L (42–960 pg/mL), which suggests that the cutoff used in the Dutch pediatric study (<33 pmol/L) is too high, giving substantial false-positive results. Other studies have also debated the use of ACTH levels in the evaluation of the HPA axis function, as it can lead to false-positive results, and recommended that the assessment of CAI should be based on 11-deoxycortisol (19, 27). Our study also confirmed the inferiority of ACTH cutoff of 33 pmol/L in the interpretation of the MTP: 21 of 46 patients who tested sufficient based on 11-deoxycortisol had peak ACTH levels < 33 pmol/L (Fig. 1b). This implies that 45.7% of our patients would have tested false-positive and would be given hydrocortisone treatment based on the ACTH cutoff used in the Dutch pediatric study (12). Some patients showed only minimal ACTH increase during MTP, whereas their 11-deoxycortisol levels strongly increased (Table 2 and Fig. 1). In 1 patient with a sufficient 11-deoxycortisol response, the ACTH level even decreased during MTP. Also, Delta ACTH correlated poorly with the 11-deoxycortisol level. These results confirm that the ACTH level during the MTP is not a reliable parameter to diagnose CAI.

An alternative explanation for the difference in test results could be that the MTP suppresses the HPA for 24 hours, whereas the sMTP, used in the Dutch pediatric study, suppresses the HPA only briefly. The administration of multiple metyrapone doses might give the patient more time to produce adequate 11-deoxycortisol levels, leading to higher 11-deoxycortisol levels. The sMTP, in which metyrapone is administered once at midnight and blood samples are collected between 8:00 am and 9:00 am, might better mimic the real-life situation in which an acute event (infection, surgery) requires a fast response of the HPA. However, this explanation seems unlikely, as CAI prevalences found by our “slow,” multiple-dose MTP are equally low as those found during ITT (in which there is an acute, short stimulation of the HPA). Furthermore, Obrynba et al, who used the sMTP (requiring a fast response), also found a low prevalence of CAI (0%) (19).

Another hypothetical explanation for the low rates of CAI in adults compared to children is that all children with CAI may have died before reaching adulthood. However, based on the incidence of PWS of around 1:16.000 live births (28) and the overall death rate in PWS of approximately 3% per year (9), this is very unlikely. Yearly, 170.000 children are born in the Netherlands (Central Bureau for Statistics, 2019) of whom 10 would be expected to have PWS. Thus, in the last 55 years, approximately 550 people with PWS are likely to have been born. If 60% of the children with PWS had CAI and all died before reaching adulthood (apart from the regular PWS mortality of 3% per year), we would expect only 220 (40% of 550) patients would be alive, of which 152 would be adults. However, in the Dutch national center of reference, over 110 adults with PWS were registered at the moment of submission of this manuscript, and we know this is far from the total Dutch adult PWS population. Therefore, the assertion that the lower rates of CAI in adults with PWS are explained solely by excess mortality due to CAI is highly unlikely.

A last theoretical explanation could be the difference in GH treatment between the Dutch pediatric study and our study. In the Dutch pediatric study all patients received GH treatment as part of a clinical trial, compared to only 28% in our study. This difference might be relevant as untreated GH deficiency may mask CAI. Low insulin-like growth factor I (IGF-I) levels result in increased expression and activity of 11β-hydroxysteroid dehydrogenase type 1 (11β-HSD1), the enzyme that converts cortisone to cortisol (29). Therefore, untreated GH deficiency may result in increased, thus falsely normal, cortisol levels. However, none of our patients had untreated GH deficiency, as all patients were tested for GH deficiency as part of regular care. Besides, we saw no differences in the peak cortisol levels between GH-treated patients and non-GH treated patients. Furthermore, in a study by Obrynba et al (19), 76% of the patients received GH treatment and none of them were diagnosed with CAI. This suggests that the low CAI prevalence that we found is not explained by untreated GH deficiency.

The review of the medical files of 645 adults with PWS attending PWS centers worldwide revealed that none of the 200 patients who underwent surgery without using hydrocortisone displayed any symptoms of hypocortisolism or adrenal crisis. This finding is in line with the results of the MTP and ITT, demonstrating that CAI is virtually absent in adults with PWS.

Only 1 patient was diagnosed with CAI, based on a peak cortisol level of 494 nmol/L during the ITT, which is just under the cutoff of 500 nmol/L. We calculated the 95% confidence interval (CI) and intra-assay coefficient of variation (VC) of the cortisol assay over 1 year to better understand the significance of this borderline-low value. The VC percentage was 6.9 and the 95% CI was 496 to 504 nmol/L; therefore, in the statistical analysis, this single patient was scored as having CAI.

To prevent further overtreatment of adults with PWS, our results will be implemented in a new guideline on the clinical management of adults with PWS. The lack of reliability of ACTH in the diagnosis of CAI will also be emphasized in the new guidelines.

In conclusion, CAI is very rare (1.2%) in adults with PWS. In order to prevent overtreatment with hydrocortisone, we advise against routine hydrocortisone administration during psychological stress, illness, or surgery in adults with PWS. In patients in whom there is a significant clinical suspicion of hypocortisolism (such as apathy, fainting, or observed hypotension during acute infections or other stressful events), we recommend testing to exclude CAI and only administer hydrocortisone if CAI is confirmed by ITT or (s)MTP.

## Additional Information:

***Disclosure Summary:*** The authors have nothing to disclose.

***Data Availability:*** All data generated or analyzed during this study are included in this manuscript or in the data repositories listed in References (25).
